# Nondestructive and noncontact evaluation of cellulose nanofiber-reinforced composites using terahertz time-domain spectroscopy

**DOI:** 10.1038/s41598-022-23865-8

**Published:** 2022-11-11

**Authors:** Atsushi Nakanishi, Naoko Kanno, Hiroshi Satozono

**Affiliations:** 1 Hamamatsu Photonics K. K., 5000 Hirakuchi, Hamakita-Ku, Hamamatsu, Shizuoka 434-8601 Japan; 2 Industrial Research Institute of Shizuoka Prefecture, 2078 Makigaya, Aoi-Ku, Shizuoka, 421-1298 Japan

**Keywords:** Applied optics, Optical techniques, Polymers

## Abstract

Cellulose nanofibers (CNFs) can be obtained from natural sources, such as plants and wood fibers. Thermoplastic resin composites reinforced with CNFs exhibit various features, including superior mechanical strength. As the mechanical properties of composites reinforced with CNFs are affected by the amount of fiber addition, it is important to determine the concentration of the CNF filler in the matrix after injection or extrusion molding. We confirmed that there was good linearity between the CNF concentration and terahertz absorption. We could distinguish the difference in the 1%-point-CNF concentration with terahertz time-domain spectroscopy. Furthermore, we estimated the mechanical properties of the CNF nanocomposites using terahertz information.

## Introduction

Cellulose nanofibers (CNFs), which generally have a diameter below 100 nm, can be obtained from natural sources, such as plants and wood fibers^1,2^. The CNFs have high mechanical strength^3^, high optical transparency^4–6^, large surface areas, and low coefficient of thermal expansion^7,8^. Thus, they are expected to be used as sustainable and high-performance materials in various applications, including electronic materials^9^, pharmaceutical materials^10^, and building materials^11^. The composites reinforced with CNFs are lightweight and exhibit high strength. Therefore, composites reinforced with CNFs may help improve the fuel economy of automobiles owing to their low weight.

To achieve high-performance properties, the homogenous distribution of CNFs in hydrophobic polymer matrices, such as polypropylene (PP), is important. Therefore, non-destructive testing methods for composites reinforced with CNFs are required. Non-destructive testing for polymer composites has been reported^12–16^. Furthermore, non-destructive testing of composites reinforced with CNFs based on X-ray computed tomography (CT) has been reported^17^. However, it is difficult to distinguish the CNFs from the matrix owing to the low-contrast images. Fluorescent labeling analysis^18^ and infrared analysis^19^ provided clear visualization of the CNFs and matrix. However, we can only obtain surface information. Therefore, these methods require cutting (destructive testing) to acquire internal information. Therefore, we propose non-destructive testing based on terahertz (THz) technology. THz waves are electromagnetic waves with frequencies between 0.1 and 10 THz. THz waves are transparent to the materials^20^. In particular, polymers and wood materials are transparent to THz waves. By using THz technology, evaluation of orientation of liquid crystal polymer^21^, and strain measurement of elastomer^22,23^ have been reported. In addition, THz sensing for wood damage caused by insect and fungal infestations in wood has been demonstrated^24,25^.

We propose the application of a non-destructive testing method to obtain the mechanical properties of composites reinforced with CNFs using THz technologies. In this study, we investigate the terahertz spectra of composites reinforced with CNFs (CNF/PP) and demonstrate the estimation of the concentration of CNFs using THz information.

## Results and discussion

**Figure 1:**
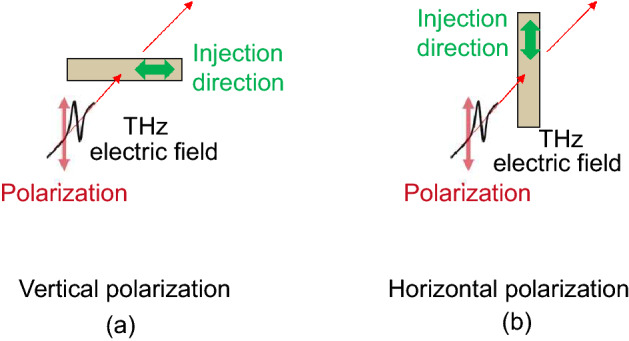
Schematic representation of the transmission measurement of composites reinforced with CNFs. (**a**) Vertical polarization; (**b**) horizontal polarization.

As the samples were prepared by injection molding, they might have had polarization dependency. Figure 1 shows the relationship between the polarization of THz waves and the direction of the samples. To confirm the polarization dependency of the CNFs, we measured their optical properties according to vertical (Fig. 1a) and horizontal polarization (Fig. 1b). Generally, compatibilizers are used to evenly disperse CNFs in the matrix. However, the influence of the compatibilizer on THz measurement has not yet been investigated. If terahertz absorption of the compatibilizer is high, transmission measurement is difficult. In addition, it is possible that terahertz optical properties (refractive index and absorption coefficient) are affected by the concentration of compatibilizers. Further, there are homo-PP and block-PP matrices of CNF composites. Homo-PP is a homopolymer of polypropylene only, and has excellent rigidity and heat resistance. Block PP, also referred to as impact copolymer, has better impact resistance than homo-PP. The composition of block-PP contains an ethylene propylene copolymer component, in addition to homo-PP, and the amorphous phase derived from the copolymer plays a role similar to that of a rubber that absorbs impact. The comparison of THz spectra has not yet been evaluated. Therefore, we first evaluated the THz spectra of PP, including that of the compatibilizer. In addition, we compared the THz spectra of the homo-PP and block-PP.

**Figure 2:**
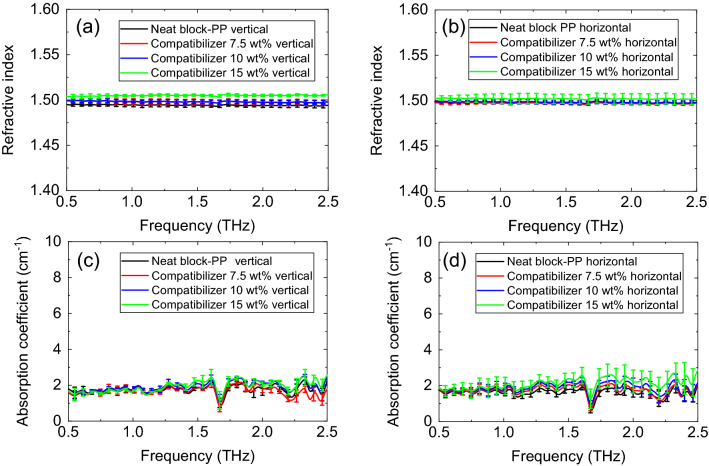
Optical properties of several PP having different compatibilizer concentration: (**a**) refractive index obtained in the vertical direction, (**b**) refractive index obtained in the horizontal direction, (**c**) absorption coefficient obtained in the vertical direction, and (**d**) absorption coefficient obtained in the horizontal direction.

We prepared a block-PP sample using maleic anhydride polypropylene (MAPP) as a compatibilizer (Umex, Sanyo Chemical Industries, Ltd.). Figure 2a,b show the THz refractive indices obtained with the vertical and horizontal polarization, respectively. Figure 2c,d show the THz absorption coefficients obtained with the vertical and horizontal polarization, respectively. As shown in Fig. 2a–d, no significant differences were observed between the THz optical properties (refractive index and absorption coefficient) with vertical and horizontal polarization. Furthermore, the compatibilizer had little influence on the THz absorption results.

**Figure 3:**
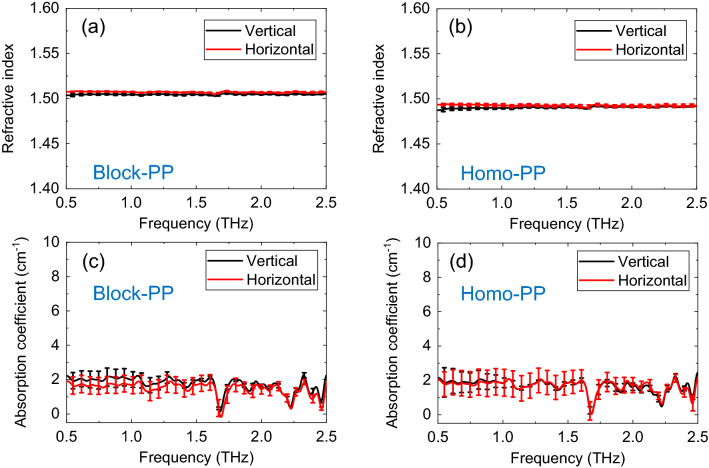
(**a**) Refractive index of block-PP, (**b**) refractive index of homo-PP, (**c**) absorption coefficient of block-PP, (**d**) absorption coefficient of homo-PP.

Subsequently, we measured neat block-PP and neat homo-PP. Figure 3a,b, respectively show the THz refractive indices of neat block-PP and neat homo-PP, obtained with vertical and horizontal polarization. The refractive indices of block-PP and homo-PP are slightly different. Figure 3c,d, respectively show the THz absorption coefficient of neat block-PP and neat homo-PP, obtained with vertical and horizontal polarization. No difference was observed between the absorption coefficients of block-PP and homo-PP.

**Figure 4:**
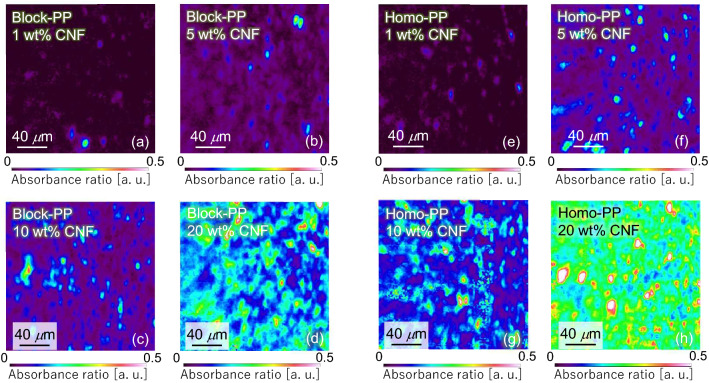
Two-dimensional distribution of CNF in PP calculated using the integrated absorbance ratio of CNF to PP: (**a**) Block-PP/1 wt% CNF, (**b**) block-PP/5 wt% CNF, (**c**) block-PP/10 wt% CNF, (**d**) block-PP/20 wt% CNF, (**e**) homo-PP/1 wt% CNF, (**f**) homo-PP/5 wt% CNF, (**g**) homo-PP/10 wt% CNF, (**h**) homo-PP/20 wt% CNF (see Supplementary Information).

Further, we evaluated the composites reinforced with CNFs. In THz measurements of composites reinforced with CNFs, it is necessary to confirm the dispersibility of the CNFs in the composite. Therefore, we first evaluated the dispersity of CNF in the composite using infrared imaging before the measurement of mechanical properties and THz optical properties. The cross-section of the sample was prepared using a microtome. Infrared images were obtained using an attenuated total reflection (ATR) imaging system (Frontier-Spotlight400, resolution 8 cm^−1^, pixel size 1.56 μm, accumulation 2 times/pixel, measurement area 200 × 200 μm, PerkinElmer). Based on the method proposed by Wang et al.^17,26^, the value obtained by dividing the peak area at 1050 cm^−1^ derived from cellulose by the peak area at 1380 cm^−1^ derived from PP was displayed as each pixel. Figure 4 shows the image that visualizes the distribution of CNFs in PP, calculated from the integrated absorbance ratio of CNFs to PP. We observed that there were a few places where the CNFs were extremely aggregated. Additionally, the coefficient of variation (CV) was calculated by applying averaging filters with different window sizes. Figure 6 shows the relationship between the window size of the averaging filter and CV.

**Figure 5:**
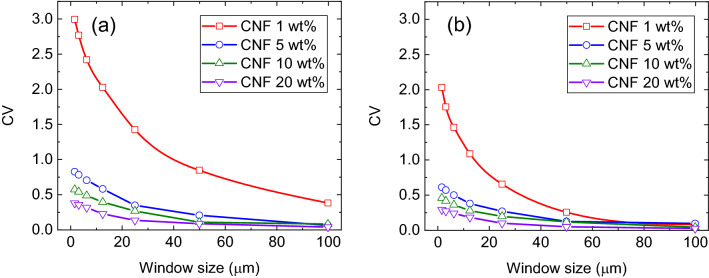
Relationship between window size of averaging filter and coefficient of the variance in integrated absorbance ratio; (**a**) Block-PP/CNF, (**b**) Homo-PP/CNF.

Although a comparison among different concentrations is inappropriate, as shown in Fig. 5, we observed that CNFs in block-PP and homo-PP exhibit close dispersity. For all concentrations, excluding 1 wt% CNF, the CV value was less than 1.0, with a gentle gradient slope. Therefore, they are considered to have high dispersity. Generally, in the case of low concentration, the CV value tends to be high for small window sizes.

**Figure 6:**
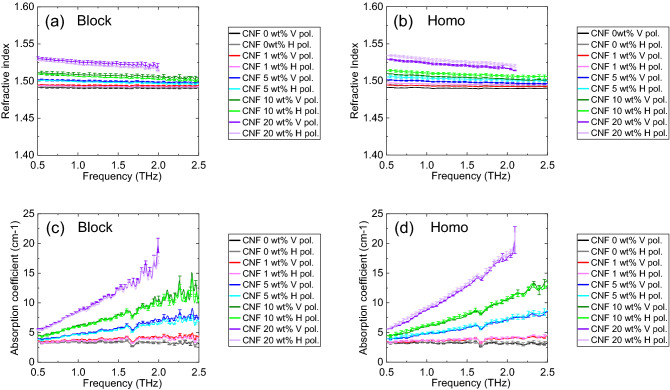
Optical properties of several PP/CNF composites with different CNF concentrations: (**a**) refractive index of block-PP/CNF, (**b**) refractive index of Homo-PP/CNF, (**c**) absorption coefficient of block-PP/CNF, (**d**) absorption coefficient of homo-PP/CNF.

We obtained the THz optical properties of the composites reinforced with CNFs. Figure 6 shows the optical properties of several PP/CNF composites with different CNFs concentrations. As shown in Fig. 6a,b, overall, terahertz refractive indices of both block-PP and homo-PP increased as the CNF concentration increased. However, it was difficult to distinguish between the 0 and 1 wt% samples because of overlapping. In addition to the refractive index, we confirmed that the terahertz absorption coefficients of both block-PP and homo-PP increased as the CNF concentration increased. Furthermore, we could distinguish between the 0 and 1 wt% samples from the result of the absorption coefficient, irrespective of the polarization direction.

**Figure 7:**
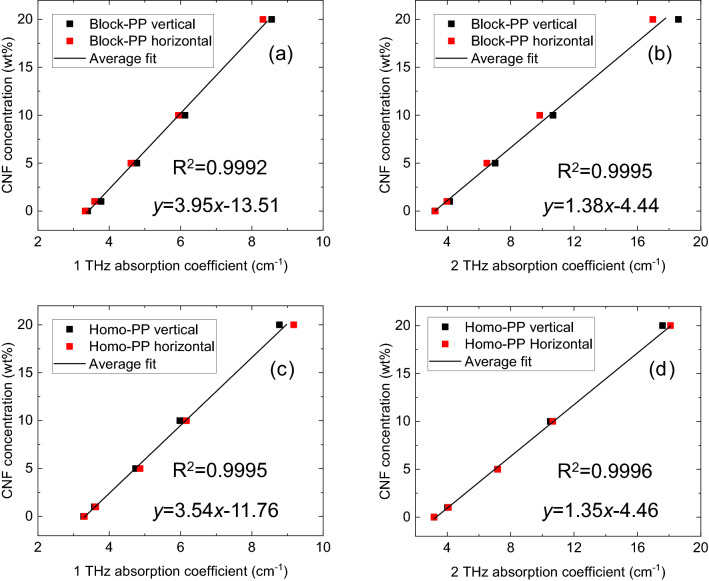
Relationship between THz absorption and CNF concentration, and linear fit obtained using the least-squares method: (**a**) Block-PP (1 THz), (**b**) Block-PP (2 THz), (**c**) Homo-PP (1 THz), (**d**) Homo-PP (2 THz). Solid line: linear fit obtained by using least-squares method.

We confirmed the linearity of the relationship between THz absorption and CNF concentration. The CNF concentration versus THz absorption coefficient is plotted in Fig. 7. The results of block-PP and homo-PP showed good linearity between THz absorption and CNF concentration. The reason for such good linearity can be explained as follows. The fiber diameter of CNF is significantly smaller than that of terahertz wavelength. Therefore, there is almost no scattering of terahertz wave in the sample. In the case of the sample with no scatter, the absorbance and the concentration have the following relationship (Beer–Lambert law)^27^.

1 $$A=\varepsilon \cdot l\cdot c,$$

where *A*, *ε*, *l*, and *c* are the absorbance, molar absorptivity, effective path length of the light through the sample matrix, and the concentration, respectively. If *ε* and *l* are constant, absorbance is proportional to the concentration.

**Figure 8:**
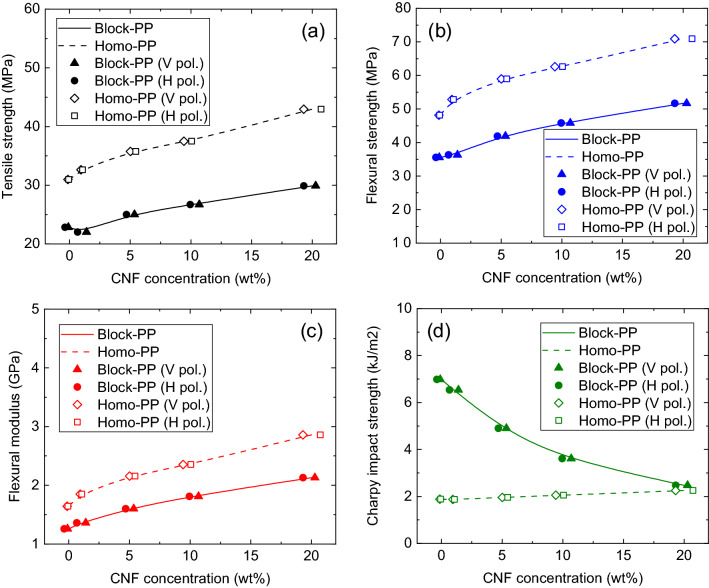
Relationship between mechanical properties and CNF concentration of block-PP (solid line) and homo-PP (dashed line), CNF concentration in block-PP estimated from the THz absorption coefficient obtained with vertical polarization (triangle), CNF concentration in block-PP estimated from THz absorption coefficient obtained with horizontal polarization (circle), CNF concentration in homo-PP estimated from THz absorption coefficient obtained with vertical polarization (diamond), CNF concentration in homo-PP estimated from THz absorption coefficient obtained with horizontal polarization (square): (**a**) Tensile strength, (**b**) flexural strength, (**c**) flexural modulus, (**d**) Charpy impact strength.

We obtained the mechanical properties of the PP/CNF composites with different CNF concentrations. For tensile strength, flexural strength and flexural modulus, the number of samples is 5 (N = 5). For Charpy impact strength, the number of samples is 10 (N = 10). These values are in accordance with the destructive testing standards to measure the mechanical strength (JIS: Japan Industrial Standard). Figure 8 shows the relationship between the mechanical properties and CNF concentration, including the estimated value, where the plots were obtained from the 1 THz calibration curves as shown in Fig. 7a,c. The curves are plotted according to the relationship between the concentration (0 wt%, 1 wt%, 5 wt%, 10 wt% and 20 wt%) and the mechanical properties. The scatter points are plotted according to the relationship between the estimated concentration and the mechanical properties at 0 wt%, 1 wt%, 5 wt%, 10 wt% and 20 wt%.

Overall, as shown in Fig. 8, the block-PP composite had better mechanical properties than the homo-PP composite. The Charpy impact strength of block-PP decreased as the CNF concentration increased. In the case of block-PP, when the PP and the masterbatch (MB) including CNF are kneaded to form a composite, the CNF forms entanglements with the PP chains; however, some PP chains are entangled with the copolymer. Further, the dispersion is inhibited. As a result, the copolymer, which absorbs impact, is inhibited by insufficiently-dispersed CNF, resulting in a decrease in impact resistance. In the case of homo-PP, CNF and PP are well dispersed, and it is thought that the CNF network structure is responsible for shock absorption.

Furthermore, the estimated CNF concentration values were on the curve showing the relationship between the mechanical properties and the actual CNF concentration. It was observed that these results were not independent of THz polarization. Therefore, we can inspect the mechanical properties of composites reinforced with CNF nondestructively using THz measurements, irrespective of THz polarization.

## Conclusion

Thermoplastic resin composites reinforced with CNFs exhibit various features, including superior mechanical strength. The mechanical properties of composites reinforced with CNFs are affected by the amount of fiber addition. We proposed the application of a non-destructive testing method to obtain the mechanical properties of composites reinforced with CNFs using THz information. We observed that the compatibilizer, which is generally added to CNF composites, had no effect on the THz measurement. We could estimate the mechanical properties of composites reinforced with CNF nondestructively using the THz absorption coefficient, irrespective of THz polarization. Moreover, this method is applicable to both CNF block-PP (CNF/block-PP) and CNF homo-PP composites (CNF/homo-PP). In this study, CNF composite samples with good dispersibility were prepared. However, depending on the manufacturing conditions, the dispersibility of CNFs in the composite can be worse. As a result, the mechanical properties of the CNF composite deteriorate due to poor dispersibility. THz imaging^28^ can be used to obtain the distribution of CNF nondestructively. However, the information in the depth direction was totaled and averaged. THz tomography technology^24^ which is used for the 3D reconstruction of the internal structure, can confirm the distribution in the depth direction. Thus, THz imaging and THz tomography provide detailed information, and we can inspect the deterioration of the mechanical properties caused by the non-uniformity of the CNFs. In the future, we plan to use THz imaging and THz tomography for composites reinforced with CNFs.

## Methods

THz-TDS measurement system was built based on a femtosecond laser (room temperature, 25 °C; humidity, 20%). The femtosecond laser beam was split into pump and probe beams by a beam splitter (BS) for terahertz wave generation and detection, respectively. The pump beam was focused on an emitter (photoconductive antenna). The generated THz beam was focused at the sample position. The THz focused beam waist was approximately 1.5 mm (FWHM). The THz beam was then transmitted through the sample and collimated. The collimated beam reached a receiver (photoconductive antenna). In the analysis method for THz-TDS measurement, the acquired terahertz electric field of both the reference and sample waveform in the time-domain are converted to complex frequency-domain electric field (*E*_ref_(ω), and *E*_sam_(ω), respectively), via fast Fourier transform (FFT). The complex transmission function *T*(ω) can be expressed using the following equation^29^

2 $$T\left(\omega \right)=\frac{{E}_{sam}\left(\omega \right)}{{E}_{ref}\left(\omega \right)}=A{e}^{i\varphi },$$

where *A* is the amplitude ratio of the sample and reference waveform, and *φ* is phase difference between the sample and reference waveform. Then, the refractive index *n*(ω) and absorption coefficient *α*(ω) can be calculated using the following equation^29^:

3 $$n\left(\omega \right)=\frac{\varphi c}{\omega d}+1,$$

4 $$\alpha \left(\omega \right)=\frac{2}{d}{\text{ln}}\left[\frac{4n\left(\omega \right)}{A{\left[n\left(\omega \right)+1\right]}^{2}}\right],$$

where *c* is speed of light, *d* is thickness of sample.

## Supplementary Information

Supplementary Information.

## Data Availability

The datasets generated during and/or analyzed during the current study are available from the corresponding author on reasonable request.
