# Supplementary material for: Nondestructive and noncontact evaluation of cellulose nanofiber-reinforced composites using terahertz time-domain spectroscopy
Source: Sci Rep. 2022 Nov 11;12:19284. doi: 10.1038/s41598-022-23865-8 (PMC9652471; doi:10.1038/s41598-022-23865-8)
Supplement: Supplementary file 1 — Supplementary Information. [file 41598_2022_23865_MOESM1_ESM.docx]

Supplementary Information

Nondestructive and noncontact evaluation of cellulose nanofiber-reinforced composites using terahertz time-domain spectroscopy

Atsushi Nakanishi^1^*, Naoko Kanno^2^, and Hiroshi Satozono^1^

^1^Hamamatsu Photonics K. K., 5000 Hirakuchi, Hamakita-Ku, Hamamatsu, Shizuoka 434-8601

^2^Industrial Research Institute of Shizuoka Prefecture, 2078 Makigaya, Aoi-ku, Shizuoka 421-1298

Correspondence to: [nakanishi@crl.hpk.co.jp](mailto:nakanishi@crl.hpk.co.jp)

**Procedure of evaluating the distribution of CNF using infrared imaging from the peak area ratio of CNF**

The distribution evaluation procedure is shown in the Fig. S1. First, we acquired the infrared spectrum at each pixel using FT-IR (Step 1). Next, as shown in Fig. S2(a), we obtained area of 1050 cm^-1^ peak (Cellulose, C-O-C stretching vibration) and 1380 cm^-1^ (PP, CH_3_ bending vibration) peak at each pixel. Then, as shown in Fig. S2(b), we obtained the two-dimensional distribution of CNF in PP, obtained from the ratio of CNF (1050 cm^-1^) to PP (1380 cm^-1^) (Step 2). Finally, we calculated standard deviation and average, and then calculated coefficient variation with different window size, as shown in Fig. S3 (Step 3). Here, we explain the calculation of coefficient variation (CV) with different window size. For example, there is peak area ratio image with 64 pixels (Fig. S3(a)). By obtaining the average value for every 2×2 pixels, we can obtain broader window size information (Fig. S3(b)). Moreover, we obtained the average value for every 4×4 pixel (Fig. S3(c)) and the average value for every 8×8 pixels (Fig. S3 (d)). In this manner, information on different window size can be obtained. CV can be calculated as

CV=SD/Ave. (1)

where SD is standard deviation and Ave. is average. As for the relationship between the window size and CV, as shown in Fig. S4, the CV approaches a constant value as the window size increase. In the case of high dispersion sample, slope would be smaller (red square). On the other hand, samples with low dispersity tend to have high slopes (black diamond). In this manner, by changing the CV and the window size and obtaining the CV, dispersibility can be evaluated not only visually but also quantitatively^1,2^.

Fig. S1. Analytical procedures

Fig. S2. (a) FT-IR spectra of CNF composites, (b) Two-dimensional distribution of CNF in PP calculated from the integrated absorbance ratio of CNF to PP.

Fig. S3. Procedure of obtaining of coefficient variation (CV) with different windows size ((a) 1 pixel, (b)2×2 pixel, (c) 4×4 pixel, (d) 8×8 pixel)

Fig. S4. Relationship between window size and CV

**Reference**

1 Okada, K., Muroga, S. & Ohshima, M. [FT-IR Imaging as a New Method to Evaluate the Additves] FT-IR imaging wo mochiita polymer composite chu no tenkazai no bunsanhyoukahou (in Japanese). *Koubunshi Ronbunshu* **75**, 212-220 (2018).

2 Wang, L. *et al.* Effect of surface modification on the dispersion, rheological behavior, crystallization kinetics, and foaming ability of polypropylene/cellulose nanofiber nanocomposites. *Compos. Sci. Technol.* **168**, 412-419, doi:10.1016/j.compscitech.2018.10.023 (2018).
